# A large scale *Plasmodium vivax- Saimiri boliviensis* trophozoite-schizont transition proteome

**DOI:** 10.1371/journal.pone.0182561

**Published:** 2017-08-22

**Authors:** D. C. Anderson, Stacey A. Lapp, John W. Barnwell, Mary R. Galinski

**Affiliations:** 1 Bioscience Division, SRI International, Harrisonburg, VA, United States of America; 2 Emory Vaccine Center, Yerkes National Primate Research Center, Emory University, Atlanta, GA, United States of America; 3 Malaria Branch, Division of Parasitic Diseases, Centers for Disease Control and Prevention, Atlanta, GA, United States of America; 4 Department of Medicine, Division of Infectious Diseases, Emory University School of Medicine, Atlanta, GA, United States of America; Bernhard Nocht Institute for Tropical Medicine, GERMANY

## Abstract

*Plasmodium vivax* is a complex protozoan parasite with over 6,500 genes and stage-specific differential expression. Much of the unique biology of this pathogen remains unknown, including how it modifies and restructures the host reticulocyte. Using a recently published *P*. *vivax* reference genome, we report the proteome from two biological replicates of infected *Saimiri boliviensis* host reticulocytes undergoing transition from the late trophozoite to early schizont stages. Using five database search engines, we identified a total of 2000 *P*. *vivax* and 3487 *S*. *boliviensis* proteins, making this the most comprehensive *P*. *vivax* proteome to date. PlasmoDB GO-term enrichment analysis of proteins identified at least twice by a search engine highlighted core metabolic processes and molecular functions such as glycolysis, translation and protein folding, cell components such as ribosomes, proteasomes and the Golgi apparatus, and a number of vesicle and trafficking related clusters. Database for Annotation, Visualization and Integrated Discovery (DAVID) v6.8 enriched functional annotation clusters of *S*. *boliviensis* proteins highlighted vesicle and trafficking-related clusters, elements of the cytoskeleton, oxidative processes and response to oxidative stress, macromolecular complexes such as the proteasome and ribosome, metabolism, translation, and cell death. Host and parasite proteins potentially involved in cell adhesion were also identified. Over 25% of the *P*. *vivax* proteins have no functional annotation; this group includes 45 VIR members of the large PIR family. A number of host and pathogen proteins contained highly oxidized or nitrated residues, extending prior trophozoite-enriched stage observations from *S*. *boliviensis* infections, and supporting the possibility of oxidative stress in relation to the disease. This proteome significantly expands the size and complexity of the known *P*. *vivax* and Saimiri host iRBC proteomes, and provides in-depth data that will be valuable for ongoing research on this parasite’s biology and pathogenesis.

## Introduction

Malaria caused by *Plasmodium vivax* is a serious neglected disease that can result in extreme morbidity and possible death [1–3]. It is characterized by approximate 48-hour cycles of reticulocyte invasion, growth, development, and release of new invasive merozoite progeny. The clinical outcome is cyclical high fever and chills, paroxysms, violent headaches, vomiting, diarrhea, and muscle aches; and the parasite can pose a particular threat to pregnant women [4]. Additional clinical observations for both *P*. *vivax* and the closely related simian malaria model species *P*. *cynomolgi* [5–7], can include an enlarged spleen, thrombocytopenia and severe anemia. This pathogen clearly poses a great public health threat, and examination of its biology, biochemistry, and pathogenesis at the molecular level is important for the development of diagnostics, therapeutics, and vaccines that can reduce disease burden [1–9].

*Plasmodium vivax* is phylogenetically distant from the pathogen causing the more lethal form of malaria, *Plasmodium falciparum* [5], thus it is important to study both species in parallel to derive the species-specific interventions important for global efforts aiming to control, eliminate and ultimately eradicate malaria [10]. About 4% of estimated global malaria cases are due to *P*. *vivax*, but outside the African continent the proportion of *P*. *vivax* infections has been reported as 41% [11]. The problem is even greater when considering this species’ dormant stage reservoir in the liver that can account for relapsing infections and asymptomatic cases that may be a source of parasites for ongoing transmission by mosquitoes, as suggested from macaque infections [6].

*Plasmodium vivax* merozoites invade reticulocytes [12–13], requiring special receptor-ligand interactions for invasion [14] with a strong predilection for invading CD71 high cells [15], which represent the youngest form of reticulocytes [16–17]. This host cell preference differs significantly from the biology of *P*. *falciparum*, which has some preference for reticulocytes but invades RBCs (red blood cells) of all ages. These differences are largely unexplored at the molecular level, and are intriguing since under normal circumstances most CD71 high reticulocytes are known to reside in the bone marrow [16]. After invasion, unlike *P*. *falciparum* trophozoite and schizont infected RBC (iRBC) that display knob-like surface structures linked to variant adhesive proteins associated with cytoadhesion and virulence [17–18], *P*. *vivax* and *P*. *cynomolgi* synthesize elaborate caveolae-vesicle complexes (CVCs), which have been observed all along the host cell membrane, with external caveolae cup-like structures and associated cytoplasmic vesicular and tubular structures [19–20] containing a variety of uncharacterized parasite-encoded membrane proteins [21]. The CVCs have been examined by electron- and immunoelectron-tomography and shown in particular to contain a highly abundant protein, PHIST/CVC-81_95_, associated with the caveolae and the cytoplasmic face of the tubules [21–22]. *Plasmodium falciparum* iRBC strongly adhere to endothelial cells deep within the venular vasculature, a process linked to severe disease [23]; endothelial cell adhesion of *P*. *vivax* iRBCs, in particular relating to the schizont stage, has only recently been proposed [24–26] and remains to be understood in detail.

Another intriguing difference across the species is that *P*. *vivax* and *P*. *cynomolgi* express members of the multigene Plasmodium interspersed repeat (pir) family, encoding PIR proteins with multiple predicted localizations (ca. 1200 in *P*. *vivax*, including the variant antigen multigene subset known as vir) [27–31], but lack the high molecular weight variant antigen families shared by *P*. *falciparum* and *Plasmodium knowlesi*, which are encoded by the var and SICAvar multigene families, respectively [32–35]. The encoded *P*. *falciparum* erythrocyte membrane protein 1 (PfEMP1) [33] and schizont infected cell agglutination (SICA) proteins are expressed at the iRBC surface and undergo switching during an immune response (reviewed in [36]). Whether *P*. *vivax* has such mechanisms, involving VIR or other proteins expressed at the surface, remains to be more deeply explored.

For both *P*. *vivax* and *P*. *falciparum*, the expressed proteome during the pathogen's life cycle in both mosquito vectors and primate hosts would be expected to differ between stages [37–39]. Each of these parasites develops in the blood over a 48-hour time period, from the ring stage of development, through a trophozoite stage in which the pathogen undergoes morphological changes, grows in size, and remodels the host iRBC, to a schizont stage in which 16–24 daughter merozoites are produced and then released into the bloodstream [40]. Experimentally derived proteomes from specific stages can be complicated by low parasitemias (typically less than 1% iRBC) in patient-isolated samples, ethical or logistical barriers drawing blood from patients, an asynchronous life cycle stage composition, and the potential for multiple strains to be present in individual patient samples, which is also likely when samples from multiple patients are pooled.

Many of these challenges can be overcome by use of nonhuman primate (NHP) models, such as the Bolivian squirrel monkey, *S*. *boliviensis* [8, 41–43], which allows optimized blood-stage *P*. *vivax* infections with blood draws timed to enrich individual life cycle stages, allowing increased association of identified proteins with individual developmental stages and disease processes. Using this animal model and 2D lc/ms/ms, and searches based on the *P*. *vivax* (Salvador-I strain; Sal-I) genome with 5459 genes [37], we have previously reported 1375 *P*. *vivax* and 3209 *S*. *boliviensis* monkey host proteins in 71–91% enriched trophozoite-stage preparations [44]. Another earlier proteomics study based on an Aotus monkey-adapted Colombian isolate reported 238 *P*. *vivax* trophozoite-stage proteins and 485 Aotus monkey host proteins in a pooled sample enriched for 70% trophozoites, and 727 *P*. *vivax* schizont-stage and 310 Aotus monkey host proteins in a pooled sample enriched for 91% schizonts [45]. Other clinical studies have captured fewer proteins. These include 16 *P*. *vivax* proteins from a single patient [46], 154 from a multi-patient pool of isolates [47], and 314 *P*. *vivax* proteins from cultured schizont-stage enriched iRBCs from a multi-patient pool also containing gametocytes [48].

With the goal of continuing to expand knowledge of the *P*. *vivax* blood-stage cycle, including modeling potential, we have developed two biological replicate trophozoite-schizont transition-stage proteomes from *P*. *vivax* (Sal-I strain) iRBCs purified from infections of two *S*. *boliviensis* monkeys. These are the first proteomes focusing on the actively growing transition period between the *P*. *vivax* trophozoite and schizont stages, including large late-stage trophozoites and early multi-nucleated (2–4) schizonts. Using methods based on four 2D lc/ms/ms runs per proteome and analysis with five database search engines [44, 49] we identified 2000 *P*. *vivax* and 3487 *S*. *boliviensis* host RBC proteins at a ~2% false discovery rate (FDR) for each search engine. We identified a number of post-translational modifications including oxidized host and parasite proteins suggestive of stress responses, as also reported in the trophozoite-enriched proteome [44], and a number of proteins that may be involved in iRBC cytoadhesion with possible survival benefits to the parasite and pathology consequences for the host. In addition, GO-term enrichment analysis of *P*. *vivax* proteins and DAVID functional annotation clustering identified host and/or pathogen enriched protein clusters that are predicted to be important, and perhaps essential, for a variety of iRBC growth and development biological and pathogenic processes.

## Materials and methods

### Animals and pathogen isolation

*Saimiri boliviensis* monkeys were acquired by the Yerkes National Primate Research Center (YNPRC), an Association for Assessment and Accreditation of Laboratory Animal Care (AAALAC) internationally-certified institution, from the Keeling Center for Comparative Medicine and Research, UT MD Anderson Cancer Center, supported by the National Institutes of Allergy and Infectious Diseases.

The animals were socially housed in pairs at the YNPRC, and all housing was in compliance with Animal Welfare Act regulations as well as the Guide for the Care and Use of Laboratory Animals. Standard procedures for splenectomy, monitoring the clinical conditions of the animals, collecting biological samples, treatment, and euthanasia were approved by Emory University’s Institutional Animal Care and Use Committee (IACUC) with the approval # YER-2003225. All nonhuman primates used in this study were provided regular environmental enrichment opportunities consisting of daily feeding enrichment, provision of manipulanda, and physical enrichment. Subjects were regularly monitored for any behavioral signs of distress by the YNPRC behavior management personnel. Animals were trained using positive reinforcement to allow blood collections from the ear without sedation.

Anesthesia was performed with Ketamine (5–10 mg/kg IM) or Telazol (3–5 mg/kg, IM). Euthanasia was performed per the recommendations of the "AVMA Guidelines for the Euthanasia of Animals: 2013 edition". Intravenous (iv) injection of barbiturates is an acceptable method of euthanasia for nonhuman primates, per these recommendations, and was approved by Emory’s IACUC. Animals are first anesthetized with either ketamine or telazol and when possible a catheter is placed in a peripheral vessel to ensure delivery. Pentobarbital 100mg/kg is injected intravenously and the animal is monitored and auscultated for cessation of heart beat and breathing.

Two independent *P*. *vivax* Sal-I blood-stage infections were initiated in *S*. *boliviensis* monkeys using procedures discussed previously [44]. Briefly, donor *S*. *boliviensis* monkeys were inoculated with cryopreserved and reconstituted *P*. *vivax* (Sal-I) iRBC monkey-adapted stocks acquired from the Centers for Disease Control and Prevention (CDC) and monitored daily; 0.5–1.0 ml of blood with a parasitemia of 0.5–1% was transferred from donor to recipient monkeys. These iRBCs had been passaged previously at the CDC in splenectomized *S*. *boliviensis* to ensure adequate peak parasitemias (typically 1–2%) and high iRBC yields; thus, splenectomies were performed in this study prior to infection to ensure comparable yields. The spleen modulates iRBC parasite variant antigen surface expression in *P*. *knowlesi* and *P*. *falciparum* infections [50–53]; but, as of yet, there is no known effect of splenectomy on the expression of any particular *P*. *vivax* protein.

The parasite density was estimated from microscopy analyses of thin blood smears, counting 2000 RBCs. Blood with respectively 0.5% and 0.9% parasitemia was collected from SB3256 and SB5115 monkeys into sodium heparin tubes and processed through ADP-glass beads and a Plasmodipur filter using standard procedures to remove platelets and white blood cells, respectively. The infected blood was then layered onto a 52% Percoll gradient to concentrate and further purify the iRBCs; platelets were less than 0.1% of the original platelet count after purification. The late-stage trophozoite/early-stage, 2–4 nucleated schizont/gametocyte differential microscopy readings for Proteomes 1 and 2 respectively were 44%/56%/0% and 40%/60%/<1%. These iRBC samples were frozen at -80 C and thawed at a later date for generation of tryptic peptides and subsequent proteomic analysis.

### Proteome analysis

Two biological replicate proteomes were analyzed. *Plasmodium vivax* (Sal-I) iRBC proteins and peptides were prepared for analysis using the FASP-II protocol [54] and analyzed using 2D SCX (strong cation exchange)/C18 RP (reversed phase) lc/ms/ms on a Thermo Scientific (San Jose, CA) LTQ-XL ETD Orbitrap mass spectrometer [44]. Four 2D lc/ms/ms runs were analyzed separately (except as noted) and identified proteins concatenated for each proteome. For Proteome 1 ca. 42 μg peptides were analyzed per run, with 12–15 salt step elutions in each run; four test C18 column runs were also included in run 1. For Proteome 2, ca. 94 μg peptides were analyzed in each of four runs, using 20–27 salt step elutions due to the larger peptide load of the SCX column. All runs used internal lock masses, selected by the mass spectrometer depending on presence of individual polysiloxane or bis(2-ethylhexyl)phthalate lock mass ions, to increase precursor ion mass accuracy, including ions at 371.101233, 391.284286, 445.120024, 519.138815 and 593.157607 m/z [55].

Database search engines used a PvP01 *P*. *vivax* reference genome [31] derived database combined with the NCBI *S*. *boliviensis* fasta protein database (both obtained from the University of Georgia Informatics Core as part of the Malaria Host-Pathogen Interaction Center, Emory University), combined with common contaminants such as the trypsin used for protein digestion, human cytokeratins, etc. [44]. Although the recently released PvP01 genome is based on a *P*. *vivax* Indonesian clinical isolate [31], it was used for analysis in preference to the Sal-I *P*. *vivax* reference genome [40], due to improved sequencing reducing fragmentation from over 2500 to 226 scaffolds, improved curation increasing the number of genes with functional attributes from 38% (Sal-I reference genome) to 58%, and improved subtelomere assembly resulting in identification of over 1200 pir genes vs. 346 in the Sal-1 genome [31].

Data analysis utilized five search engines. Andromeda (v. 1.2.0.14, embedded in Maxquant software v. 1.2.0.18) [56] used precursor and fragment ion uncertainties of 13 ppm and 0.8 Da respectively; for each proteome all four 2D lc/ms/ms runs were included in a single analysis with a maximum peptide FDR (false discovery rate) of 0.2 and maximum protein FDR of 0.1; identified proteins were then selected to a maximum protein PEP (posterior error probability) of 0.02. Mascot [57] v. 2.3.02 with Mascot Distiller v. 2.4.2.0 included proteins up to a protein FDR of 2.17%, and used Percolator peptide spectrum match scoring [58]. SEQUEST [59] utilized Percolator peptide scoring embedded in Thermo Proteome Discoverer v. 1.3.0.339 software, with protein PEP maximally 2% calculated using custom Excel macros based on the Protein Prophet algorithm without the mixture model [60, 44]. Two sets of runs were analyzed, one using only carbamidomethyl-cys and met sulfoxide as variable modifications, the second using a more extensive set of oxidative modifications (below). The fourth search engine utilized was Crux with Percolator scoring [61]; proteins were accepted to a maximal protein q value of 0.02. The fifth search engine was MSGF Plus [62], using Mascot to convert Thermo.raw files to mgf format, and Proteowizard [63] to convert output.mzid files to.pepXML files for analysis by IDPicker 3 and IDPAssemble [64]. The maximum MSGF Plus protein FDR was 2%. For protein identification, searches were conducted with a precursor ion tolerance of 13 ppm and product ion tolerance of 0.8 Da, required a single unique peptide for identification [65] and full tryptic specificity, and used a maximum of two missed tryptic cleavage sites.

Identification at least twice by a search engine (similar to [66]) was required for consideration of the protein's function when assessing *P*. *vivax* or *S*. *boliviensis* biology. Different search engines used different algorithms for protein grouping; proteins are thus presented as individual proteins independent of groups, with information on individual search engine results presented in S1 Table, S2 Table, S3 Table and S4 Table. Pseudogenes were not listed as identified proteins.

To streamline the modification identification protocol used previously for trophozoite-enriched proteomes [41], post-translational modifications (mainly oxidized) were identified in a single SEQUEST database search with Percolator scoring. Variable modifications included optional monoisotopic mass additions of 15.995 Da (oxidation) or 31.990 Da (dioxidation) for C, F, H, M, W and Y, 44.985 Da (nitration) or 60.980 (nitrohydroxylation) for F, H, W and Y, 47.985 Da (trioxidation) for C, W and Y, and 57.021 (carboxamidomethylation) for C, with a maximum of 3 identical modifications per peptide. Only b and y ions were considered for identification; peptides accepted as oxidatively modified had a Percolator [58]—calculated posterior error probability of 0.01 or less, a delta score of 1.00 (i.e. the modified peptide was the only identification for the peptide-spectral match found in the search), and a search engine rank of 1. For oxidative modifications, both trophozoite-schizont transition proteomes were analyzed, and the trophozoite proteomes [44] re-analyzed, using a recently released PvP01 genome database [31] to allow direct comparisons based on the best annotated *P*. *vivax* genome to date. Identifications by SEQUEST from separate searches for oxidative modifications and basic modifications (M oxidation, C carbamidomethylation) were combined.

### Functional annotation

Functional annotation for piecharts, of proteins identified at least twice by a search engine, was from PlasmoDB [67], Uniprot [68], NCBI Entrez [69], CDD [70], PubMed or the primary literature, KEGG [71], or InterPro [72]. When proteins were associated with multiple functions, the apparent most prominent functional category was listed. The function of human homologs of *S*. *boliviensis* proteins is generally listed when available. The source of the functional annotation, when not obvious from the protein description, is listed in a separate annotation column. The "transcription" functional category includes transcription factors, RNA polymerase complex proteins, and proteins involved in RNA polyadenylation, capping and splicing, and RNA transport to the cytoplasm. The "translation" category includes ribosome assembly proteins, ribosomal proteins, and proteins involved in elongation on tRNA and termination.

GO term enrichment (relative to the whole genome) for *P*. *vivax* proteins used PlasmoDB [67] release 31, included proteins identified at least twice by a search engine, included GO biological process, molecular function, and cell compartment, and included the default P-value cutoff of 0.05. The Benjamini-Hochberg FDR [73] is listed for each enriched cluster.

For analysis of *S*. *boliviensis* identified proteins, we used Database for Annotation, Visualization and Integrated Discovery (DAVID) v6.8 Bioinformatics Resources [74–75]. *Saimiri boliviensis* proteins were first blasted against the human genome using BlastP [76] with the top-ranked human protein saved for analysis. A list of human protein accession numbers was then submitted to DAVID for medium stringency classification, against a background of the Homo sapiens genome, using the annotation terms GOTERM_MF_ALL, GOTERM_BP_ALL, GOTERM_CC_ALL, KEGG_PATHWAY, BIOCARTA, BBID, COG ontology, UP_KEYWORDS, and OMIM disease. Individual annotation clusters were generally named by the top term or a consensus term. Clusters are listed with Enrichment Score, and the Benjamini-Hochberg FDR for the top term of the cluster.

## Results

### Protein identifications

**Fig 1:**
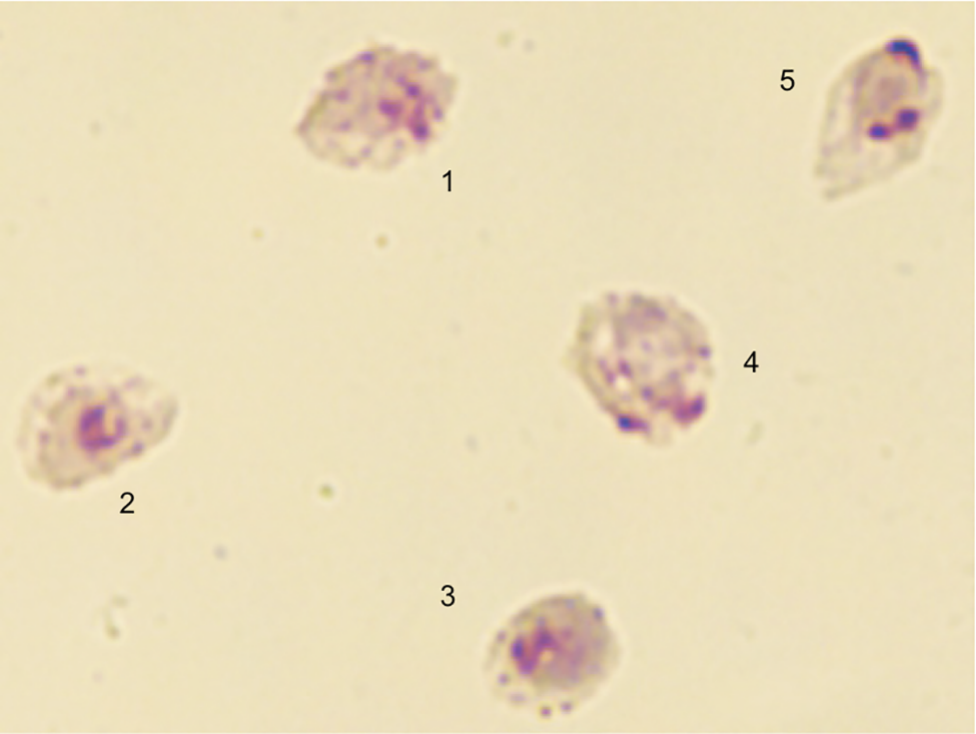
Giemsa-stained *P*. *vivax* iRBC isolated from biological replicate 1. Thin smears were prepared from Percoll-gradient enriched iRBC, and show early nucleated schizonts (iRBC 1, 4 and 5) and large late-stage trophozoites (iRBC 2 and 3).

Fig 1 illustrates the representative iRBC stages isolated from biological replicate 1, which contained 44% trophozoites, 56% schizonts, and no apparent gametocytes. The morphology of individual iRBCs illustrates the nature of the trophozoite/schizont transition stages used to derive Proteome 1, as iRBCs 1, 4 and 5 are early nucleated schizonts, while iRBCs 2 and 3 represent large late-stage trophozoites. Biological replicate 2, used to obtain Proteome 2, contained 40% trophozoites, 60% schizonts, and <1% gametocytes.

A summary of proteins identified in Proteomes 1 and 2 is listed in S1 Table (Proteome 1) and S2 Table (Proteome 2). S3 Table lists *S*. *boliviensis* and *P*. *vivax* proteins combined from both proteomes; in total, 2000 *P*. *vivax* and 3487 *S*. *boliviensis* proteins were identified by at least one search engine.

The relative abundances of *P*. *vivax* and *S*. *boliviensis* proteins are listed in S4 Table, calculated using all 8 2D lc/ms/ms runs from both proteomes, as the exponentially multiplied protein abundance index emPAI [77] by Mascot [57]. The most abundant *P*. *vivax* protein, glyceraldehyde-3-phosphate dehydrogenase (GPDH), is ca. 15-fold more abundant than the same (but distinct) *S*. *boliviensis* protein. Other abundant *P*. *vivax* proteins include a number of additional glycolytic enzymes (enolase, phosphoglycerate kinase, lactate dehydrogenase, fructose 1,6-bisphosphate aldolase, pyruvate kinase, triose phosphate isomerase, phosphoglycerate mutase), the four core histones comprising a nucleosome, two heat shock proteins (HSP), ribosomal protein P2, elongation factor 1 alpha, the CVC protein PHIST/CVC-81_95_ (PVP01_0119200) [22], and an endoplasmic reticulum (ER) calcium binding protein proposed as a target of anti-malarial endoperoxides in *P*. *falciparum* [78]. The most abundant *S*. *boliviensis* proteins include five hemoglobin chains, ribosomal and ubiquitin-related proteins, the Ig superfamily hypothetical protein C17orf99 homolog, cytoplasmic actin, flavin reductase, two carbonic anhydrase enzymes, and the proinflammatory cytokine IL-36 beta. Nine different complement proteins are identified at least twice by a search engine; of these three (C9, factor I, C1Q) have a relative abundance in the range of 0.15–0.21, and six (factor B, C4-A, C3, C5, C7, C4b binding protein alpha) have a relative abundance in the range of 0.02–0.07).

### Protein functional categories

**Fig 2:**
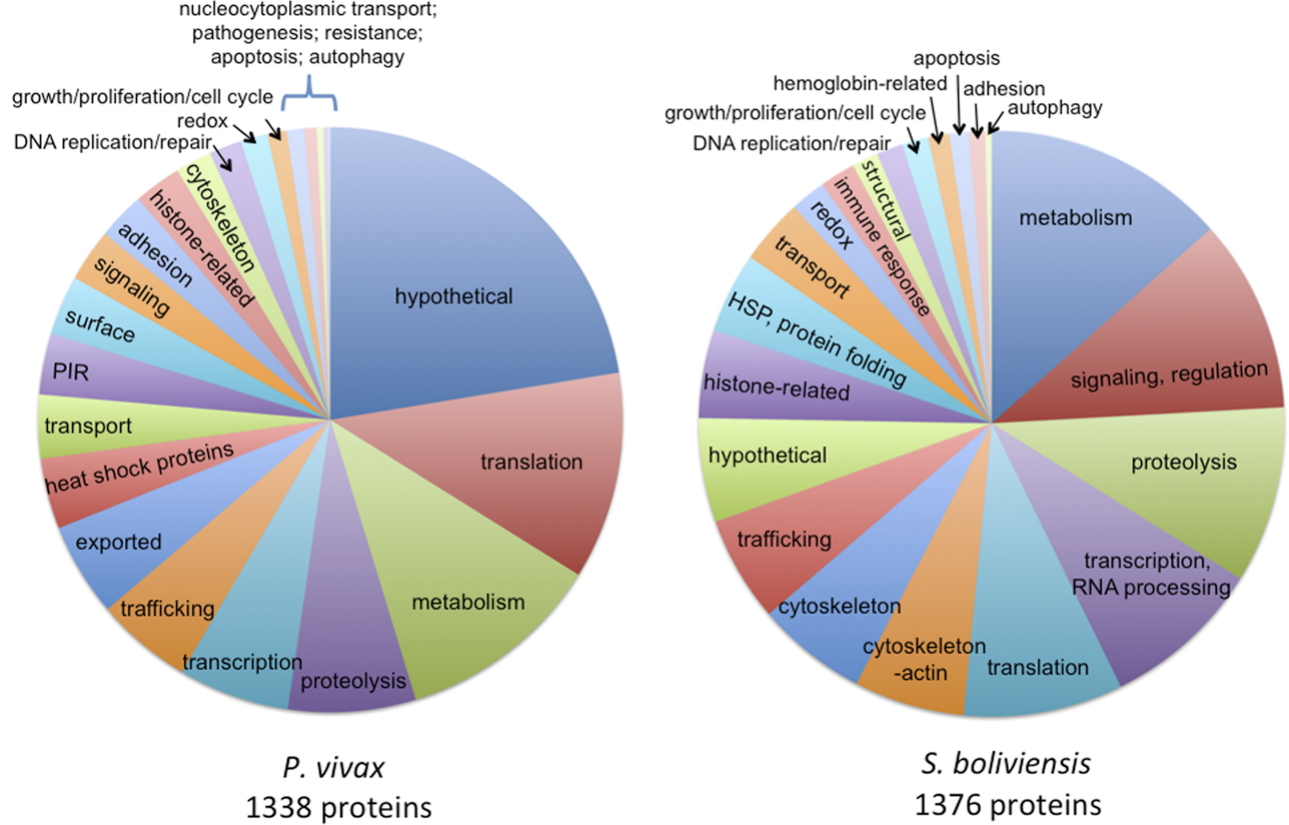
Functional categories of *P*. *vivax* and *S*. *boliviensis* trophozoite-schizont transition iRBC proteins. Major categories for both organisms include metabolism, translation, transcription, proteolysis and trafficking. Over 25% of *P*. *vivax* proteins (hypothetical and PIR proteins) have no annotated function. Numerous cytoskeletal proteins, particularly actin-related, are identified for *S*. *boliviensis*. Details of individual proteins, each identified more than once by a database search engine in the two combined biological replicate proteomes, are in S5 Table. These pie charts are similar to those of trophozoite-stage iRBC proteins [44], however the *P*. *vivax* hypothetical protein fraction has decreased. New *P*. *vivax* categories include PIR/VIR proteins and Plasmodium exported proteins.

Fig 2 shows pie charts for one overview of functional categories of proteins identified at least twice by a search engine in the analysis of the two trophozoite-schizont transition proteomes; this includes a subset totaling 1338 *P*. *vivax* and 1376 *S*. *boliviensis* proteins. Individual protein functional categories are listed in S5 Table, along with 15 *P*. *vivax* and 64 *S*. *boliviensis* proteins annotated as mitochondrial proteins. The largest *P*. *vivax* category, including ca. 22% of this proteome, consists of hypothetical proteins without functional annotation. Other major categories include translation, metabolism, proteolysis, transcription and trafficking. Two additional significant categories include Plasmodium exported proteins and PIR/VIR proteins. *Saimiri boliviensis* iRBC proteins were more evenly divided among a number of categories, the largest being metabolism, signaling, proteolysis, transcription and translation. About half of the identified cytoskeletal proteins were linked to actin. For both organisms, the functional pie charts are similar to those from our previously reported trophozoite-enriched stage [44]; use of the new PvP01 reference genome for database searches here may have decreased the fraction of *P*. *vivax* hypothetical proteins. Identification of PIR/VIR proteins was also substantially increased (to 45) compared to analysis with the Sal-I database, which could reflect stage or isolate specific differences though also likely reflects the improved assembly and annotation of the telomeres in this reference sequence

### Protein functional clustering using PlasmoDB and DAVID

To further characterize overall function of proteins expressed in Proteomes 1 and 2, we used GO term enrichment analysis, implemented in PlasmoDB [66], to analyze *P*. *vivax* proteins (Table 1), and DAVID v. 6.8 [74–75] functional annotation clustering to analyze *S*. *boliviensis* proteins (Tables 2 and 3).

**Table 1:** GO-term enrichment analysis of *P*. *vivax* proteins in proteomes 1 and 2.

GO ID	GO Term, Proteome 1	Fold enrichment	Benjamini FDR	GO ID	GO Term, Proteome 2	Fold enrichment	Benjamini FDR
	**Biological Process**				**Biological Process**		
GO:0006096	glycolytic process	3.96	0.010	GO:0006890	retrograde vesicle-mediated transport, Golgi to ER	3.79	0.038
GO:0006090	pyruvate metabolic process	3.6	0.013	GO:0009064	glutamine family amino acid metabolic process	3.79	0.043
GO:0006414	translational elongation	3.05	0.019	GO:0006096	glycolytic process	3.03	0.035
GO:0051169	nuclear transport	2.86	0.010	GO:0006090	pyruvate metabolic process	2.76	0.040
GO:0015991	ATP hydrolysis coupled proton transport	2.83	0.020	GO:0006414	translational elongation	2.62	0.040
GO:0009166	nucleotide catabolic process	2.64	0.017	GO:0015991	ATP hydrolysis coupled proton transport	2.44	0.041
GO:0006184	GTP catabolic process	2.55	0.019	GO:0016052	carbohydrate catabolic process	2.44	0.041
GO:0006457	protein folding	2.45	0.001	GO:0006511	ubiquitin-dependent protein catabolic process	2.27	0.009
GO:0045454	cell redox homeostasis	2.38	0.019	GO:0006413	translational initiation	2.17	0.040
GO:0006511	ubiquitin-dependent protein catabolic process	2.14	0.017				
GO:0006413	translational initiation	2.17	0.040				
	**Molecular Function**				**Molecular Function**		
GO:0070003	threonine-type peptidase activity	3.81	0.005	GO:0070003	threonine-type peptidase activity	3.28	0.009
GO:0002161	aminoacyl-tRNA editing activity	3.67	0.046	GO:0051082	unfolded protein binding	2.22	0.017
GO:0016209	antioxidant activity	3.42	0.040	GO:0004812	aminoacyl-tRNA ligase activity	2.15	0.013
GO:0051020	GTPase binding	3.3	0.043	GO:0003743	translation initiation factor activity	2.11	0.024
GO:0019829	cation-transporting ATPase activity	2.75	0.036				
GO:0051082	unfolded protein binding	2.73	0.007				
GO:0003743	translation initiation factor activity	2.28	0.036				
	**Cell Component**				**Cell Component**		
GO:0005852	eukaryotic translation initiation factor 3 complex	4	0.009	GO:0044433	cytoplasmic vesicle	3.79	0.006
GO:0000785	chromatin	3.85	0.023	GO:0005798	Golgi-associated vesicle	3.79	0.017
GO:0005839	proteasome core complex	3.81	0.003	GO:0030137	COPI-coated vesicle	3.79	0.024
GO:0005773	vacuole	3.67	0.039	GO:0000785	chromatin	3.32	0.027
GO:0015935	small ribosomal subunit	3.3	0.023	GO:0005839	proteasome core complex	3.28	0.008
GO:0030135	coated vesicle	3.05	0.023	GO:0033176	proton-transporting V-type ATPase complex	2.76	0.033
GO:0005798	Golgi-associated vesicle	2.93	0.044	GO:0005794	Golgi apparatus	2.59	0.022
GO:0033176	proton-transporting V-type ATPase complex	2.8	0.039	GO:0015935	small ribosomal subunit	2.53	0.043
GO:0005794	Golgi apparatus	2.32	0.038	GO:0031410	cytoplasmic vesicle	2.41	0.022
GO:0031410	cytoplasmic vesicle	2.8	0.011				

**Table 2:** DAVID-derived *S*. *boliviensis* functional annotation clusters in proteomes 1 and 2.

Enrichment Score	Proteome 1	Benjamini top term	Enrichment Score	Proteome 2	Benjamini top term
	**vesicles, trafficking**			**vesicles, trafficking**	
154.35	extracellular exosome	9.10E-178	80.19	extracellular exosome	5.20E-92
52.94	intracellular organelle	1.50E-58	14.81	membrane-bounded organelle	1.80E-29
25.72	organelle lumen	6.80E-37			
3.71	cytoplasmic vesicle	3.10E-06			
3.01	endocytic vesicle lumen	2.50E-04			
2.36	nucleocytoplasmic transport	1.40E-05			
2.09	exocytosis	5.60E-04			
2.08	autophagy	3.00E-02			
2.04	RNA export from nucleus	5.60E-05			
	**cytoskeleton**			**cytoskeleton**	
50.2	non-membrane-bounded organelle/cytoskeleton	5.40E-60	11.07	non-membrane-bounded organelle/cytoskeleton	2.60E-17
29.67	adherens junction	9.50E-53	17.49	adherens junction	1.10E-25
19.14	cytoskeleton	1.80E-27	7.91	cortical cytoskeleton	7.00E-10
11.06	cortical cytoskeleton	1.80E-13	6.14	spectrin-associated cytoskeleton	1.30E-07
7.32	actin cytoskeleton	2.80E-18	5.89	contractile fiber	5.30E-09
6.16	Intermediate filament	4.90E-13	3.89	actin cytoskeleton	6.20E-10
5.91	microtubules	4.40E-07	2.95	microtubule cytoskeleton	1.80E-05
5.58	contractile fiber	4.40E-10	2.08	dynein complex	2.20E-04
5.27	actin filament bundle	5.90E-06			
4.43	spectrin-associated cytoskeleton	9.90E-07			
2.83	myosin complex	4.10E-05			
2.01	actin-myosin filament sliding	5.50E-06			
	**oxidation**			**oxidation**	
7.67	oxidation-reduction process	5.30E-12	5.14	oxidation-reduction process	1.20E-06
5.85	antioxidant activity	2.70E-08	4.78	response to oxidative stress	3.00E-07
3.28	cell redox homeostasis	5.70E-05	3.77	Oxidoreductase	6.10E-04
2.99	hydrogen peroxide metabolic process	1.10E-06	2.94	glutathione metabolic process	5.90E-03
2.85	Oxidation	2.40E-07			
2.83	glutathione metabolic process	1.10E-03			
2.4	response to oxidative stress	3.00E-07			
	**pathogen**			**pathogen**	
31.5	symbiosis, encompassing mutualism through parasitism	2.80E-34	9.7	symbiosis, encompassing mutualism through parasitism	4.20E-11
12.2	Viral nucleoprotein	3.00E-15	2.62	inclusion body assembly	3.00E-03
7.84	Pathogenic Escherichia coli infection	1.80E-11			
2.16	establishment of viral latency	3.10E-02			
3.22	viral genome replication	1.40E-04			
3.25	inclusion body assembly	2.00E-03			
	**homeostasis**			**homeostasis**	
4.81	erythrocyte homeostasis	9.10E-09	2.88	tissue homeostasis	1.60E-04

**Table 3:** DAVID-derived *S*. *boliviensis* functional annotation clusters in proteomes 1 and 2.

Enrichment Score	Proteome 1	Benjamini top term	Enrichment Score	Proteome 2	Benjamini top term
	**protein folding**			**protein folding**	
14.5	protein folding	3.80E-15	5.32	protein folding	6.80E-16
3.58	protein stabilization	4.90E-06	2.71	response to unfolded protein	3.40E-06
3.26	chaperonin-containing T-complex	2.80E-06	2.62	inclusion body assembly	3.00E-03
3.25	inclusion body assembly	2.00E-03			
2.31	unfolded protein response	1.50E-03			
2.19	protein folding in endoplasmic reticulum	1.10E-04			
2.14	chaperone mediated protein folding	4.10E-02			
	**macromolecular complexes**			**macromolecular complexes**	
25.4	cellular macromolecular complex assembly	1.20E-32	11.1	macromolecular complex assembly	4.40E-13
25.1	ribonucleoprotein complex	1.10E-69	11.7	Proteasome	3.30E-26
13.2	spliceosome	6.60E-28	4.31	proteasome regulatory particle	5.50E-11
9.97	nucleosome	1.80E-15	3.66	regulation of proteasomal catabolic process	1.30E-09
6.5	ribosome assembly	7.80E-10	3.02	Protease	1.10E-03
4.48	proteasome accessory complex	1.10E-08	2.04	chromatin	2.00E-04
2.64	protein localization to chromatin	3.10E-04			
2.3	lysosome	2.00E-03		**metabolism**	
	**metabolism**		9.00	small molecule metabolic process	5.90E-19
11.99	ATP metabolic process	1.40E-17	6.93	phosphorus metabolic process	2.20E-09
11.09	energy derivation by oxidation of organic compounds	3.30E-17	6.85	cofactor metabolic process	5.10E-12
9.42	small molecule metabolic process	2.20E-16	4.86	Protein biosynthesis	6.20E-10
5.27	tricarboxylic acid cycle	3.20E-06	3.6	aldehyde/NADP metabolic process	3.10E-07
4.54	NADP metabolic process	1.70E-06	2.6	Porphyrin biosynthesis	2.80E-02
2.62	ribonucleotide metabolic process	1.50E-10	2.47	regulation of transmembrane transport	1.40E-04
2.43	hemoglobin complex	2.40E-05	2.43	Purine biosynthesis	7.90E-04
2.29	glycogen metabolism	5.50E-03	2.41	hemoglobin complex	1.60E-04
2.25	pyruvate metabolism	8.40E-04	2.39	cysteine metabolic process	9.90E-04
2.21	DNA metabolic process	5.30E-03	2.19	L-ascorbic acid metabolic process	2.20E-03
	**RNA**			**RNA**	
46.96	poly(A) RNA binding	1.10E-69	7.89	poly(A) RNA binding	1.80E-10
9.08	regulation of RNA stability	1.30E-23	3.33	translation elongation	1.00E-04
5.48	regulation of translation	2.10E-08	2.56	Aminoacyl-tRNA synthetase	3.20E-03
2.99	regulation of RNA splicing	1.20E-05	2.22	translational initiation	1.10E-02
2.59	translation elongation factor activity	4.10E-03			
2.09	aminoacyl-tRNA ligase activity	4.20E-07			
	**cell death**			**cell death**	
4.19	negative regulation of programmed cell death	9.20E-06	3.44	regulation of cell death	1.50E-04
3.42	mitochondrion	2.90E-08	3.32	regulation of neuron apoptotic process	1.90E-03
3.42	apoptotic mitochondrial changes	2.40E-06	2.44	apoptotic mitochondrial changes	1.70E-03
3.31	regulation of apoptotic signaling pathway	3.40E-05			
3.25	regulation of neuron apoptotic process	2.30E-03			

Table 1 lists some representative *P*. *vivax* non-redundant GO-term enrichment clusters for both proteomes, with two-fold or higher enrichment. The entire set of clusters is listed in S7 Table (Proteome 1) and S8 Table (Proteome 2). A number of enriched clusters are common to both biological replicate proteomes, as expected, including glycolysis and pyruvate metabolism; translation initiation, elongation, the small ribosomal subunit and aminoacyl tRNA ligase activity; the ubiquitin-proteasome system; unfolded protein binding, and chromatin. Another set of related clusters common to both proteomes includes intracellular vesicle systems (e.g. cytoplasmic vesicles, Golgi-associated vesicles and the Golgi apparatus, coated vesicles), and related processes such as V-type ATPase activity. Proteome 1 uniquely also included clusters for antioxidant activity and cell redox homeostasis.

Table 2 presents results from DAVID-based protein annotation clustering of *S*. *boliviensis* proteins. Each individual entry represents a cluster that is at least two-fold enriched, with an enrichment score (relative to the human genome) and the Benjamini-Hochberg FDR listed for the top term of each cluster. The clusters are grouped into sets of similar clusters, representing vesicles and trafficking, the cytoskeleton, oxidation, pathogen processes, and homeostasis. The most highly enriched single cluster includes extracellular exosomes for both Proteomes 1 and 2, with an 80-154-fold enrichment. The cytoskeleton set of clusters consists of 8–12 individual clusters for Proteomes 1 and 2, including enrichment of e.g. the actin cytoskeleton, microtubules, spectrin-associated cytoskeleton, and adherens junctions. Analysis of both proteomes highlights a set of oxidation-related clusters, which includes response to oxidative stress and glutathione metabolic processes, and in the more oxidized Proteome 1, antioxidant activity, cell redox homeostasis, hydrogen peroxide metabolic processes and oxidation clusters were prominently identified. Both proteomes contain the ~10–30 fold enriched cluster "symbiosis encompassing mutualism through parasitism"; Proteome 1 also contains clusters for pathogenic *E*. *coli* infection and three viral infection-related clusters. Details of the clusters are presented in S9 Table.

Table 3 presents additional *S*. *boliviensis* enriched protein annotation clusters related to protein folding and the response to unfolded proteins, macromolecular complexes (e.g. the proteasome (both proteomes), and ribosome, spliceosome, and nucleosome in Proteome 1), metabolism, RNA-related clusters (particularly translation and splicing), and a set of cell death clusters including apoptotic mitochondrial changes, regulation of neuron apoptotic processes, and regulation or negative regulation of (programmed) cell death. Additional clusters in Proteome 1 include regulation of apoptotic signaling pathway and the mitochondrion. Details of each individual cluster are presented in S9 Table.

### Cytoadhesion proteins

S10 Table lists *P*. *vivax* proteins from the trophozoite-schizont transition proteomes that are annotated as, or predicted to be potentially involved in cytoadhesion, and identified at least twice by a search engine. These include four merozoite rhoptry proteins [45, 79–80] among five proteins associated with host-cell binding or invasion, including an erythrocyte binding protein and a Duffy receptor-binding protein [14]. An additional 29 MAAP-predicted adhesins [81] were identified, as were 45 PIR/VIR proteins, some of which may have functions other than adhesion, e.g. antigenic variation.

Sixteen *S*. *boliviensis* proteins are also annotated as involved in cytoadhesion, including the transferrin receptor (CD71 antigen, here identified as transferrin receptor protein 1), shown to be an important marker (when at high levels) of reticulocytes preferentially invaded by *P*. *vivax* [15] and *P*. *yoelli* [16]. Other proteins may directly mediate cell-cell adhesion, such as thrombospondin-3, fermitin family homolog 3, or protocadherin 10, or may be less directly involved in cell adhesion, such as CECAM 18, ankyrin-3 or talin-2.

### Protein oxidation and nitration

We previously reported significant oxidation and nitration of both *P*. *vivax* and *S*. *boliviensis* trophozoite-stage enriched proteins in two biological replicates [44], including as examples *S*. *boliviensis* hemoglobin, actin, and the *P*. *vivax* CVC protein PHIST/CVC-81-95 [22]. Table 4 compares these observations to the same proteins in the transition Proteomes 1 and 2 identified here. Datasets for all four proteomes were analyzed, using the newly released *P*. *vivax* reference genome sequence [31] for residue oxidation and nitration in a single Sequest database search, as discussed in the methods section. Both of the hemoglobin alpha and beta chains are significantly oxidized in all four proteomes. Oxidation of cytoplasmic actin and the PHIST/CVC-81-95 protein are more variable, particularly in the trophozoite proteomes; details of high-confidence trophozoite-schizont transition oxidized peptides, and their cognate proteins, are contained in S6 Table. With two exceptions, all four proteins have a significant number of oxidized residues in both the trophozoite and trophozoite-schizont transition proteomes.

**Table 4:** Hemoglobin, actin, PHIST/ CVC-81_95_ protein oxidized/nitrated residues.

Proteome:	Trophozoite 1	2	Trophozoite-Schizont Transition 1	2
Protein	# oxidized residues^1^^,^^2^			
*S*. *boliviensis* Hb β chain	17	20	36	24
*S*. *boliviensis* Hb α chain	18	14	37	28
*S*. *boliviensis* actin, cytoplasmic^1^	20	0	10	6
*P*. *vivax* PHIST/CVC-81_95_ (PVP01_0119200)	6	0	8	6

^1^ oxidized and nitrated residues include met, cys, his, trp, tyr and phe (see methods section)

but exclude met sulfoxide as met can be oxidized in solutions exposed to air.

^2^ peptide PEP ≤ 0.01

Table 5 examines the oxidized amino acids present in high-confidence tryptic peptides from both Proteomes 1 and 2, comparing the fraction of oxidized residues to residues in a control *Mycobacterium smegmatis* proteome prepared using the same FASP-II protocol, electrosprayed under identical conditions [44], and analyzed with the identical Sequest/Percolator workflow as the two trophozoite-schizont proteomes. Proteome 1 generally contains a higher fraction of oxidized residues than Proteome 2. With two exceptions (singly oxidized met- which is readily produced by dissolved oxygen in solution [82], and trp), oxidized met, tyr, trp, cys, phe and his occur at higher levels in Proteome 1 than in the control proteome. Proteomes 1 and 2 have higher levels of nitrated or nitrohydroxylated residues than the control proteome, in which neither modification is observed under these analytical conditions. Proteome 2 has several modifications at lower levels than the control proteome (e.g. singly oxidized met, tyr, and trp), but most highly oxidized residues (his, phe, cys, tyr, met) are more prevalent in Proteome 2 than in the control proteome. Excluding proteins with only methionine oxidized to met sulfoxide, and now analyzed identically to the transition proteomes, trophozoite Proteome 1 had the largest number of oxidized host (274) and parasite (72) proteins, compared to trophozoite Proteome 2 (18 host and 12 parasite highly oxidized proteins). Combined, transition trophozoite-schizont Proteomes 1 and 2 have 74 oxidized *S*. *boliviensis* proteins or subunits with at least one high confidence oxidized residue and 144 oxidized *P*. *vivax* proteins or subunits.

**Table 5:** Trophozoite-schizont transition proteome oxidative modifications^1^.

	Proteome 1		Proteome 2		Control	
peptides:	5028		4377		3043	
**aa, mod**	#	**Fraction**	#	**Fraction**	#	**Fraction**
**met**	1255		1449		604	
unmodified	133	0.1060	899	0.6204	48	0.0795
O	994	0.7920	491	0.3389	522	0.8642
O_2_	128	0.1020	109	0.0752	34	0.0563
**tyr**	2162		2282		1137	
unmodified	1990	0.9204	2240	0.9816	1121	0.9859
O	81	0.0375	13	0.0057	14	0.0123
O_2_	12	0.0056	7	0.0031	2	0.0018
O_3_	13	0.0060	15	0.0066	0	0.0000
NO_2_	60	0.0278	6	0.0026	0	0.0000
NO_2_OH	6	0.0028	1	0.0004	0	0.0000
**trp**	180		295		401	
unmodified	122	0.6778	254	0.8610	233	0.5810
O	25	0.1389	25	0.0847	136	0.3392
O_2_	17	0.0944	16	0.0542	26	0.0648
O_3_	11	0.0611	0	0.0000	6	0.0150
NO_2_	2	0.0111	0	0.0000	0	0.0000
NO_2_OH	7	0.0389	0	0.0000	0	0.0000
**cys**	457		796		149	
unmodified	20	0.0438	1	0.0013	0	0.0000
CAM^2^	403	0.8818	784	0.9849	149	1.0000
O	16	0.0350	0	0.0000	0	0.0000
O_2_	8	0.0175	2	0.0025	0	0.0000
O_3_	10	0.0219	9	0.0113	0	0.0000
**phe**	2639		2659		1870	
unmodified	2513	0.9523	2611	0.9819	1846	0.9872
O	68	0.0258	30	0.0113	21	0.0112
O_2_	32	0.0121	16	0.0060	3	0.0016
NO_2_	13	0.0049	1	0.0004	0	0.0000
NO_2_OH	13	0.0049	1	0.0004	0	0.0000
**his**	1922		2600		1259	
unmodified	1813	0.9433	2539	0.9765	1246	0.9897
O	42	0.0219	26	0.0100	10	0.0079
O_2_	41	0.0213	33	0.0127	3	0.0024
NO_2_	16	0.0083	2	0.0008	0	0.0000
NO_2_OH	10	0.0052	0	0.0000	0	0.0000

^1 ^All peptides have an observed precursor ion mass less than 5 ppm from the theoretical mass;

^2^ carboxamidomethyl

## Discussion

We have identified 2000 *P*. *vivax* and 3487 *S*. *boliviensis* trophozoite-schizont transition proteins by at least one of five search engines, using two biological replicates, expanding identifications from previous *P*. *vivax* trophozoite and schizont proteomes, and enhancing the modeling potential of this parasite’s biology by the broad research community. All data has been deposited in PlasmoDB. In addition to analysis of each biological replicate with four separate 2D lc/ms/ms runs, and use of higher amounts of tryptic peptides than our *P*. *vivax* trophozoite proteome published in 2015 with 1375 *P*. *vivax* and 3209 *S*. *boliviensis* proteins [44], analysis here using five search engines [83–84, 66, 44] has aided this process. The current analysis also benefits from use of a database derived from a more comprehensive reference genome sequence [31], which may reduce the percentage of unannotated proteins from 33% in [44] to 22% here.

Interestingly, the *S*. *boliviensis* host transferrin receptor protein 1 (transferrin receptor, or CD71 antigen) was identified in the trophozoite-enriched as well as the current proteomes, a point worth noting given *P*. *vivax*’s known preference of invading CD71 high reticulocytes and open questions surrounding the parasite-host receptor-ligand and invasion requirements [15, 16, 85]. The late trophozoite represents the growing parasite with dramatic host cell modifications, including the development of CVCs; and the early, 2–4 nucleated schizont stage represents the beginning of parasite division and the development of new progeny, the infectious merozoite forms of the parasite. In the future, a comparable proteome of the matured *P*. *vivax* schizont stage–with up to 16 merozoites–would be valuable for the characterization of late-stage schizont proteins including a predominance of merozoite proteins that are important for egress from their host RBCs and invasion of reticulocytes to propagate the blood-stage infection as well as development of new sexual stage forms for infection of the Anopheles mosquito vector and continued transmission. While genome and transcriptome studies (37–39, 31) can predict this parasite species and stage-specific proteomes and help generate hypotheses, proteomic studies provide the evidence of protein expression. Future time course analyses with integrated omics (e.g. transcriptome, proteome, lipidome, and metabolome) will help develop a deeper appreciation of the parasite’s unique biology, over ~48 hours between the invasion, takeover, and destruction of the reticulocyte with the release of new merozoite progeny.

The most abundant *P*. *vivax* trophozoite-schizont stage protein is GPDH, ca. 15-fold more abundant than the same (but distinct) *S*. *boliviensis* protein, and ca. 6–10 fold more abundant than additional *P*. *vivax* glycolytic enzymes. Elevated levels of the *P*. *vivax* glycolytic enzymes are consistent with 50–100 fold increased glucose consumption in *P*. *falciparum*-infected erythrocytes [86], as glycolysis is this pathogen's sole source of energy [87]. Enzymes such as GPDH can have additional roles beyond metabolism, including functions in oxidative stress and apoptosis [87]. Other abundant *P*. *vivax* proteins include heat shock proteins (HSPs), histones H4 and H2B, and elongation factor-1-alpha, as well as the *P*. *vivax* caveolae vesicle complex PHIST protein CVC-81-95 reported originally to be a main protein of the CVCs [21–22]. The *P*. *falciparum* schizont-stage proteome also includes as highly abundant proteins GPDH and other glycolytic enzymes, histones H4 and H2B, three HSPs and elongation factor-1-alpha [88].

As expected, the most abundant *S*. *boliviensis* protein identified is hemoglobin, whose alpha subunit is ca. 160-fold above levels of the next most abundant non-hemoglobin protein, GPDH. Two host carbonic anhydrases are identified here at relatively high levels. Carbon dioxide is essential for iRBC growth, pyrimidine biosynthesis and control of intracellular pH [89]; carbonic anhydrase facilitates CO_2_ transport across the RBC plasma membrane. This parasite enzyme has been noted as a potential malaria drug target [89–90]; the presence of significant levels of host iRBC carbonic anhydrases may be a consideration for development of such inhibitors. Identification of nine complement components may be relevant given observations of complement fixation in *P*. *vivax* malaria [91] and complement activation by the surface of *P*. *falciparum* iRBC [92–93] and *P*. *knowlesi* [94].

### Protein functional clusters

Protein function is presented using several methods. Piecharts give a broad overview of the proteome and highlight some significant areas such as hypothetical proteins, exported proteins, and PIR proteins less readily observed by *P*. *vivax* GO-term enrichment. DAVID functional annotation clustering and PlasmoDB-based GO-term enrichment focus on gene sets enriched for particular molecular functions, biological processes or cellular locations.

Little is known about the restructuring of the host reticulocyte and the development of numerous caveolae vesicle complexes (CVCs), which include parasite-encoded proteins positioned all along the *P*. *vivax* infected host cell membrane [20–22]. In addition to the *P*. *vivax* PHIST protein CVC-81_95_ [22], other CVC proteins [21] may well be represented among the hypothetical proteins identified and further research will be required to confirm their presence, locations and functions. Pertinent to these distinctive biological features, with much yet to be discovered on the functions of the CVCs with their complex array of tubules and vesicles [22], results from both PlasmoDB and DAVID highlight intracellular vesicles and processes, and organelle and macromolecule trafficking. These data suggest that these processes are part of both the host and parasite encoded iRBC biology. The highest DAVID enrichment score is obtained for extracellular exosomes, perhaps reflecting exosome-mediated loss of cell surface molecules in the transition from reticulocytes to mature RBCs (or iRBCs) [15, 95–96]; *P*. *yoelii* exosomes have in fact been shown to contain parasite proteins, and these may be involved in cell-cell communication and immune modulation [97]. Proteins important for vesicle trafficking have also been identified in an uninfected murine reticulocyte proteome [98]. Retrograde vesicle transport from the Golgi apparatus to the ER, identified as a *P*. *vivax* cluster from Proteome 2, is important for secreted proteins; 69 exported proteins, related proteins, or Plasmodium exported proteins were also identified. This retrograde transport can involve COPI-coated vesicles, the Golgi apparatus, and Golgi-associated vesicles [99], all of which are enriched *P*. *vivax* GO-term clusters, as well as protein folding in the ER. Gautier et al. [100] identified 33 Golgi proteins that partitioned 20% or more into cultured human reticulocytes after orthochromatic cell enucleation. Organelle trafficking can involve autophagy, important for elimination of mitochondria during reticulocyte development [101–103], apicoplast maintenance and protein/organelle trafficking [104], and apicoplast-mediated [105–106] iRBC cell death by atypical autophagy [107]. ROS and RNS can be linked to autophagy [108] and may connect autophagy and apoptosis [109].

A second area of protein annotation cluster enrichment in Proteomes 1 and 2 includes DAVID-derived host cytoskeleton-related clusters, e.g. the actin cytoskeleton, microtubules, the spectrin-associated cytoskeleton, contractile fibers, and adherens junctions. When orthochromatic erythroblasts enucleate to form reticulocytes, cytoskeletal proteins, including the spectrin cytoskeleton, microtubules, myosin and actin [110] are retained in the reticulocyte. The cytoskeleton significantly rearranges during reticulocyte maturation, which includes proteasome degradation of tubulin and actin [111]; both DAVID and PlasmoDB also identify enriched proteasome or ubiquitin-dependent protein catabolism clusters here. Although not enriched at least twofold in GO-term clusters, a number of *P*. *vivax* cytoskeletal proteins (e.g. alpha and beta tubulin, dynein, myosin A and E, several actins and actin-like proteins) were also identified.

A third set of enriched clusters includes host protein DAVID-identified cell death-related clusters in both proteomes, e.g. regulation of (programmed) cell death, apoptotic mitochondrial changes, and regulation of neuron apoptotic processes. Reticulocytes are enucleated; however anucleate cells can undergo forms of apoptosis. Anucleate platelet apoptosis has some features in common with nucleated cell apoptosis, e.g. expression of pro- and anti-apoptotic proteins, depolarization of the mitochondrial inner membrane and cytochrome c release, surface exposure of phosphatidyl serine, activation of caspase 3, platelet shrinkage and fragmentation [112]. Red blood cells, without a nucleus or mitochondria, undergo programmed cell death (eryptosis) similar to apoptosis, in which the cells shrink, undergo membrane blebbing, with phosphatidylserine exposed at the surface to enable phagocytosis by macrophages [113–114]. Eryptosis can be induced by a variety of xenobiotics, Ca^2+^ entry, ceramide formation, caspase stimulation, energy depletion, and oxidative stress [114]. *Plasmodium falciparum* infection of erythrocytes can induce oxidative stress and trigger eryptosis [115]; premature eryptosis of iRBC before Plasmodium release may protect against malaria [115]. Reticulocytes contain mitochondria, which are eliminated by the time they mature to normocytes [98, 101, 116]. We identified ca. 15 host proteins annotated as involved in apoptosis including phospholipid scramblase 1, which may expose phosphatidyl serine on the surface of iRBC, allowing iRBC phagocytosis by macrophages [117]. We also identified the *P*. *vivax* anti-apoptotic protein bax inhibitor 1, which could inhibit bax-mediated apoptosis signaling and premature iRBC death, as well as 64 host and 15 parasite mitochondrial proteins. It is possible that some of these proteins are contaminants from platelets not completely removed during iRBC purification; however in a proteome of human reticulocytes isolated from cell culture, 6 apoptosis proteins partition after orthochromatic erythroblast enucleation into the reticulocyte at a level of 5% or higher, as do 105 mitochondrial proteins and 198 nuclear or nucleolar proteins [100]. It is thus possible that a form of apoptosis or eryptosis may be relevant to *P*. *vivax* iRBC biology and the normal host response to parasitism.

### Protein oxidation and nitration

We evaluated oxidized *P*. *vivax* and *S*. *boliviensis* proteins in the two transition proteomes, as before for a trophozoite proteome [44], as they are indicative of possible stress reactions. To control for protein oxidation due to sample preparation and handling, we compared the levels of oxidized residues to those in a proteome from cultured Mycobacterium smegmatis [44], which does not contain food vacuoles; generally, higher levels of oxidized residues were observed in both transition proteomes when compared to the control proteome. The same FASP-II sample preparation protocol, and electrospray conditions, were used for all three proteomes. Peptides from each proteome were electrosprayed from silica capillary columns at or below 2.1 kV, avoiding higher (3.5 kV) spray voltages associated with corona discharge-associated oxidation from stainless steel capillary columns [118], which were not used here. Although the concentration of all oxidizing species will be significantly diluted upon addition of 4% SDS for cell lysis, it is possible that some oxidative modifications could occur after cell lysis if oxidizing species continue to be produced.

Proteome 1 appears to be more extensively oxidized than Proteome 2 (Table 5), which in turn appears to be mostly more oxidized than the control proteome. The identification by DAVID of host *S*. *boliviensis* functional annotation clusters for response to oxidative stress, antioxidant activity, cell redox homeostasis, oxidation, hydrogen peroxide metabolic processes, glutathione metabolic processes, and *P*. *vivax* cell redox homeostasis and antioxidant enriched GO-term clusters in the more heavily oxidized Proteome 1 (Table 5), suggests the presence of iRBC proteins to counteract oxidative stress in these iRBC. The observation of a variety of oxidized protein residues, compared to the control proteome, is consistent with observations of an internal oxidizing environment in *P*. *falciparum* iRBC [40, 119]. As such, these are worth further consideration on the path to understanding *P*. *vivax* pathogenesis.

**Fig 3:**
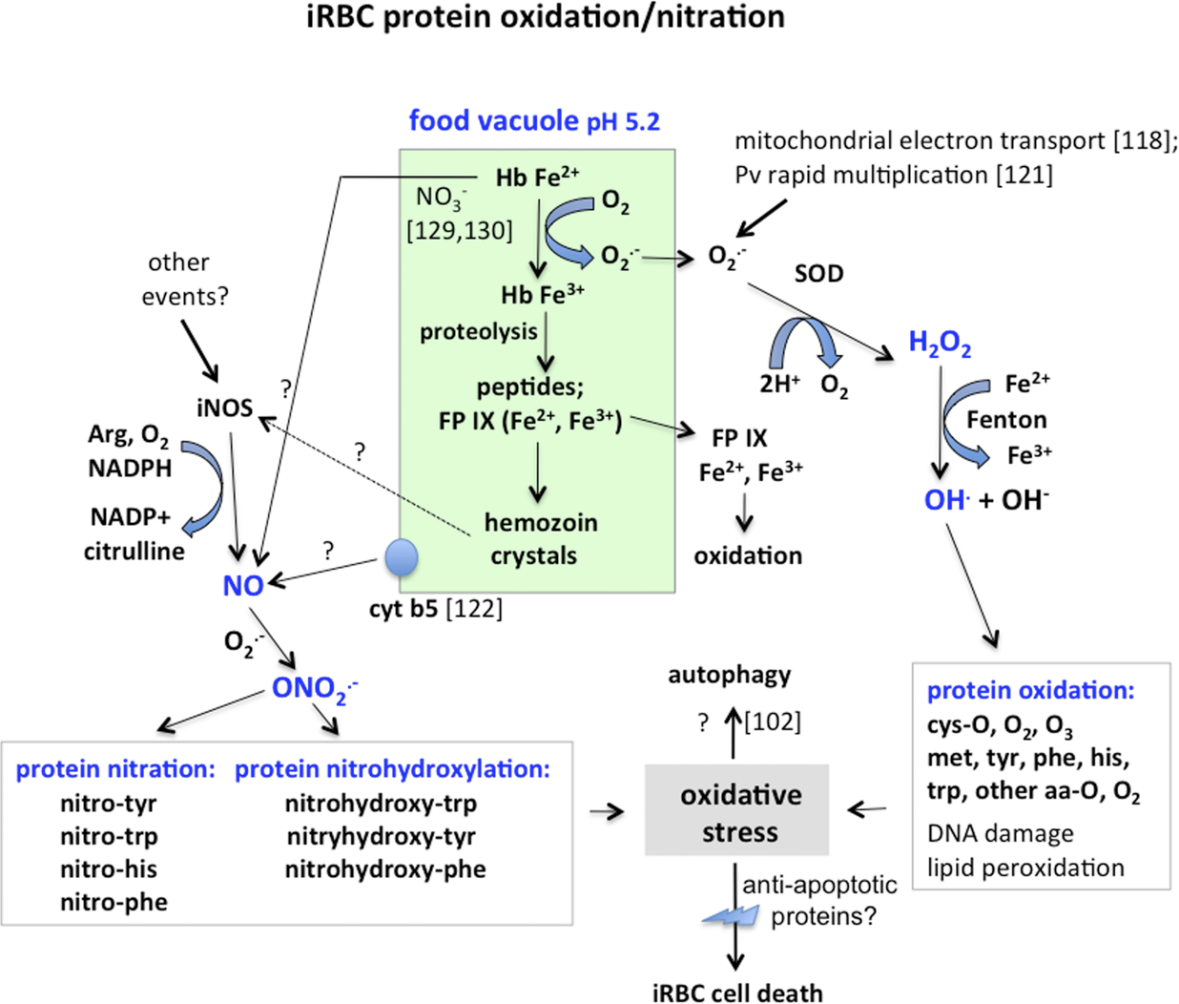
Summary of *P*. *vivax* iRBC oxidative reactions [40, 119–122]. Hemoglobin (Hb) oxidation in food vacuoles may be central to generation of superoxide and production of hydrogen peroxide (H_2_O_2_), leading to production of hydroxyl radicals and observed protein sidechain oxidations. Food vacuoles may also be central to production of nitric oxide (NO) [123–124], which could be produced by additional pathways [125–128], leading to reaction with superoxide, generation of peroxynitrite (ONO_2_), and nitration and nitrohydroxylation of additional protein sidechains. This protein oxidation and nitration may lead to or be part of iRBC oxidative stress. FP IX (ferri- or ferroprotoporphyrin IX), iNOS, inducible nitric oxide synthase; SOD, superoxide dismutase; Cyt b5, Cytochrome b5.

Since protein nitration or oxidation in this context is a chemical process, the rate of reaction may follow second order kinetics, thus the rate, and extent of reaction at a fixed time, will depend on the concentration of both the protein side chains accessible to a reaction, and the concentration and reactivity of oxidizing/nitrating species; this may bias identifications to abundant proteins (e.g. hemoglobin or actin) or those close to the site of ROS (reactive oxygen species) or RNS (reactive nitrogen species) generation. Highly reactive hydroxyl radicals with a short lifetime (ca. 2 ns) will derivatize proteins only within ca. 20 Å of their source [120]. Our observations provide a list of hydroxylated proteins in this category and may suggest their presence close to or within food vacuoles or other cellular sources (Fig 3). The presence of antioxidants or enzymes degrading ROS (e.g. peroxiredoxins [121], superoxide dismutases, glutathione and thioredoxin systems [122]) will also affect the process of protein oxidation/nitration.

ROS can be generated by degradation of hemoglobin in food vacuoles [121], the high metabolic rate of the multiplying parasite and increased mitochondrial respiratory chain production of superoxide [122], and the host immune response [119]. The low food vacuole pH of ca. 5.2 [119] is associated with oxidation of oxyhemoglobin ferroprotoporphyrin IX to ferriprotoporphyrin IX and production of superoxide, which can spontaneously dismutate in food vacuoles to H_2_O_2_ and O_2_ [119]; in the cytosol superoxide is degraded to H_2_O_2_ and O_2_ by superoxide dismutase. The Fenton reaction, utilizing Fe^2+^, produces OH^.^ radicals from H_2_O_2_ [125], which can oxidize protein residues. Oxidation of some proteins, such as *S*. *boliviensis* actin at Y240, may in fact result in altered function, as noted earlier [44].

The significant protein nitration we observe could result from induction of the pathophysiological NO/ONOO cycle, some elements of which are observed here [125]. However the source(s) of nitric oxide (NO) and peroxynitrite are unclear; due to the high reactivity of peroxynitrite, NO generation should be local [129]. Nitric oxide and RNS are present in *P*. *falciparum* trophozoite food vacuoles [123–124]; their production could be due, in *P*. *falciparum*, to a food vacuole-associated cytochrome b5 [123–124]. *Plasmodium vivax* trophozoite and schizont iRBC also contain food vacuoles; thus, the *S*. *boliviensis* cytochrome b5 identified in the transition proteome could potentially contribute to NO generation. Nitric oxide can also be generated by reaction of deoxyhemoglobin with the erythrocyte cytosolic metabolite nitrite [130–131], which is present at ca. 300 nM levels in uninfected erythrocytes [123].

It is also possible that NO production is initiated in *P*. *vivax* iRBC by other events. The hemoglobin catabolite hemozoin, produced in iRBC food vacuoles, increases iNOS (inducible nitric oxide synthase) in macrophages [126]. Induction of iNOS [127] in iRBC could result in high levels of NO, and reaction of the NO with superoxide to produce peroxynitrite (Fig 3), which in turn can nitrate and nitrohydroxylate [120, 132] proteins; and, in some cells iNOS can induce apoptosis [133].

### Cytoadhesion

Virulence of *P*. *falciparum* is due in part to strong iRBC adhesion to the endothelium in the deep venular vasculature [134]. *Plasmodium vivax* iRBC may adhere to endothelial and other cell types under static and flow conditions [24], adhere to ICAM-1 under flow conditions via specific VIR proteins [28]; or undergo (cytoadhesive) rosetting with uninfected RBC [135]. Lower levels of *P*. *vivax* schizont iRBC observed in the blood may be due to enhanced schizont adhesion compared to other stages of asexual development [26]. As iRBC cytoadherence could utilize both host and pathogen proteins, we have examined the *P*. *vivax* trophozoite-schizont transition proteome for proteins that might be involved in adhesion. Enhanced sequestration of *P*. *falciparum* iRBC may involve altered expression of endothelial cell adhesion receptors linked to oxidative stress [119]. In view of our observation of highly oxidized host and parasite proteins, observation of a number of host protein enriched functional clusters involved in oxidative stress or the response to oxidation, and parasite protein GO term clusters enriched in proteins involved in antioxidant activity or cell redox homeostasis, it is possible that oxidative stress and cytoadhesion are linked for *P*. *vivax* as well.

The proteome identified here contains 45 PIR/VIR proteins identified at least twice by a search engine; we recognize that details of the expressed VIR proteins (e.g. sequences outside of identified tryptic peptides) could differ between the PvP01 isolate and Sal-I strain, and that a Sal-I genome with improved subtelomere sequencing may enable the identification of additional VIR proteins. Individual VIR proteins could have multiple functions, including immune evasion by adherence to spleen barrier cells to escape clearance by spleen macrophages [27, 136], adhesive rosette formation and/or invasion [137], and antigenic variation [50–52, 27–28, 84, 136]. A definitive role in adhesion will require further tests of individual VIR proteins. Other cytoadhesive iRBC parasite proteins include a number of erythrocyte binding or reticulocyte invasion proteins, or *P*. *vivax* analogs of *P*. *falciparum* erythrocyte binding or adhesion proteins, as well as 29 additional MAAP-predicted adhesins [81]. Surface proteins with roles in immune evasion and/or cytoadhesion, are of interest also as possible vaccine candidates [136]. A number of identified *S*. *boliviensis* proteins are implicated in cell adhesion processes, including thrombospondin-3, fermitin family homolog 3, protocadherin 10-like, vitronectin, talin-2, neuroplastin, retinoschisin and PDZ domain-containing protein 2. Thus both host and/or parasite proteins may potentially be involved in adhesion.

## Conclusions

In this paper, we have examined the *P*. *vivax* trophozoite-schizont transition iRBC proteome using a new *P*. *vivax* reference genome, identifying 2000 *P*. *vivax* and 3487 host *S*. *boliviensis* proteins. Over 22% of *P*. *vivax* proteins still have no functional annotation, highlighting a large gap in the basic molecular understanding of *P*. *vivax* biology. Oxidation and nitration observed in the trophozoite stage-enriched proteome appear also to be present at the transition to the schizont stage and could reflect iRBC oxidative stress. GO-term enrichment analysis highlighted a number of parasite metabolic processes and molecular functions including glycolysis, translation and protein folding, cell components such as ribosomes, proteasomes and the Golgi apparatus, and a number of vesicle- and macromolecule/organelle trafficking-related clusters. DAVID analysis of *S*. *boliviensis* proteins included cytoskeletal actin-, tubulin- and spectrin-related clusters, oxidative processes and response to oxidative stress, vesicle and trafficking-related clusters including exosomes and exocytosis, macromolecular complexes such as the proteasome and ribosome, metabolism, translation, and cell death. Host and parasite proteins potentially involved in cell adhesion were also identified. These identifications may provide details for a deeper understanding of *P*. *vivax* biology as well as hypotheses for the functional involvement of individual proteins in iRBC biology, parasitism, immunity and pathogenesis.

## Supporting information

S1 Table Proteome 1 protein identifications. This table shows Proteome 1 identifications using 5 different search engines. *P*. *vivax* proteins are indicated by accession numbers beginning with PvP01_ and *S*. *boliviensis* proteins are identified by accession numbers consisting of nine numbers. Crux did not include protein annotations with identifications. XID is the identification number of the protein; Xrep is the number of search engines identifying the protein. Proteins identified by all 5 engines are listed in the worksheet "5x of 5x"; proteins identified by only one of 5 engines are in the worksheet "1x of 5x" etc. Avg. Xrep is the average number of times a search engine identified a protein from within the four individual 2D lc/ms/ms runs for each proteome. For Maxquant, all four individual 2D lc/ms/ms runs were analyzed at once (giving more identifications than analyzing each run separately), thus Avg. Xrep is 1 for all proteins. For Sequest, all four 2D lc/ms/ms runs were analyzed twice (see the methods section), thus Avg. Xrep is maximally 8 for Sequest.
(XLS)

S2 Table Proteome 2 protein identifications. This table shows Proteome 2 identifications using 5 different search engines. *P*. *vivax* proteins are indicated by accession numbers beginning PvP01_, and *S*. *boliviensis* proteins are identified by accession numbers consisting of nine numbers. XID is the identification number of the protein; Xrep is the number of search engines identifying the protein. Proteins identified by all 5 engines are listed in the worksheet "5x of 5x"; proteins identified by only one of 5 engines are in the worksheet "1x of 5x" etc. Xrep is the number of search engines identifying the protein; Avg. Xrep (as for S1 Table) is the average number of times a search engine identified a protein from within the four individual 2D lc/ms/ms runs for each proteome. Crux did not include protein annotations with identifications.
(XLS)

S3 Table Combined proteomes 1 and 2 protein identifications. This table contains all of the *P*. *vivax* and *S*. *boliviensis* proteins identified at least once by a search engine, obtained by combining S1 Table and S2 Table. For this table Xrep refers to identification in Proteome 1 and/or Proteome 2.
(XLS)

S4 Table Relative abundance of *P*. *vivax* and *S*. *boliviensis* proteins identified by Mascot, calculated using emPAI. This table contains the relative abundance of *P*. *vivax* and *S*. *boliviensis* proteins, for all proteins identified by Mascot using the exponentially multiplied protein abundance index (emPAI) [65]. Values were averaged for identifications from both proteomes 1 and 2. Xrep is the number of times the protein was identified by Mascot in the total of 8 2D lc/ms/ms runs included from both proteomes.
(XLS)

S5 Table Functional categorization of identified proteins. This table shows the functional categorization of proteins identified in combined trophozoite-schizont transition Proteomes 1 and 2 for both *P*. *vivax* and *S*. *boliviensis*. Proteins included have been identified at least twice by a search engine in the combined proteomes. The functional categories are listed to the right of the proteins, which are identified by accession number. Xrep represents identification of the protein in one or both proteomes; Avg. Xrep is the average number of times a single search engine identified a protein from within the four individual 2D lc/ms/ms runs for each proteome, and must be at least 2. The protein descriptions from the database used for identification are also listed. Some details from additional annotation (e.g. from Uniprot) are listed in the Annotation column. Proteins are placed into broad functional categories of interest, e.g. cytoskeleton(-related) as indicated. More than one functional category may be listed when this is supported by available information.
(XLS)

S6 Table Sequest-identified proteins/protein groups containing high confidence oxidized peptides. This table lists all Sequest-identified proteins containing at least one high confidence (Percolator posterior error probability of 0.01 or less) oxidized peptide, in trophozoite-schizont transition Proteomes 1 and 2. Proteins from both *P*. *vivax* and *S*. *boliviensis* are listed in layer 1, and details of peptide Sequest scores, and modifications of individual peptides, are listed in layer 2 for each proteome. Proteins containing only methionine oxidized to met sulfoxide, which can occur in solutions exposed to air, are excluded. Proteome 1 contains 52 oxidized *S*. *boliviensis* and 83 oxidized *P*. *vivax* proteins or subunits, Proteome 2 has 38 oxidized *S*. *boliviensis* and 86 oxidized *P*. *vivax* proteins or subunits; the combined proteome contains 74 different oxidized *S*. *boliviensis* and 143 different oxidized *P*. *vivax* proteins or subunits.
(XLS)

S7 Table GO term enrichment for *P*. *vivax* proteins from proteome 1. Enrichments were obtained using PlasmoDB. The table lists all GO terms, including GO_biological process, GO_molecular function, and GO_cellular component, enriched for *P*. *vivax* proteins from proteome 1.
(XLS)

S8 Table GO term enrichment for *P*. *vivax* proteins from proteome 2. Enrichments were obtained using PlasmoDB. The table lists all GO terms, including GO_biological process, GO_molecular function, and GO_cellular component, enriched for *P*. *vivax* proteins from proteome 2.
(XLS)

S9 Table DAVID Annotation cluster enrichment for *S*. *boliviensis* proteins from proteomes 1 and 2. This table presents results for all DAVID-derived annotation clusters of *S*. *boliviensis* proteins identified at least twice by a search engine. Each cluster is composed of a set of similar GO terms (e.g. biological process or BP, molecular function or MF, cell component or CC). The overall cluster is generally named for the top term or a consensus of individual terms; each GO term has a p-value and Benjamini-Hochberg false discovery rate calculated by DAVID 6.8. Annotation clusters for Proteomes 1 and 2 are in separate tabs.
(XLS)

S10 Table Cytoadhesion-related proteins. This table lists *P*. *vivax* and *S*. *boliviensis* proteins identified in trophozoite-schizont transition Proteomes 1 and/or 2 at least twice by a search engine, that are either annotated as involved in cell adhesion, or predicted to be adhesins [78]. We also included 45 VIR proteins, which mainly have no known function but have been hypothesized to be involved in adhesion, and/or trafficking, signaling, or antigenic variation/immune evasion.
(XLS)
